# Reduced Theta Connectivity during Set-Shifting in Children with Autism

**DOI:** 10.3389/fnhum.2013.00785

**Published:** 2013-11-14

**Authors:** Sam M. Doesburg, Julie Vidal, Margot J. Taylor

**Affiliations:** ^1^Department of Diagnostic Imaging, The Hospital for Sick Children, Toronto, ON, Canada; ^2^Neurosciences & Mental Health Program, The Hospital for Sick Children Research Institute, Toronto, ON, Canada; ^3^Department of Medical Imaging, University of Toronto, Toronto, ON, Canada; ^4^Department of Psychology, University of Toronto, Toronto, ON, Canada; ^5^Paris Descartes University, Paris, France; ^6^UMR, Centre National de la Recherche Scientifique 3521, Paris, France

**Keywords:** ASD, autism, neural synchrony, neural oscillation, magnetoencephalography, set-shifting, executive function, functional connectivity

## Abstract

Autism spectrum disorder (ASD) is a characterized by deficits in social cognition and executive function. An area of particular difficulty for children with ASD is cognitive flexibility, such as the ability to shift between attentional or response sets. The biological basis of such deficits remains poorly understood, although atypical development of structural and functional brain connectivity have been reported in ASD, suggesting that disruptions of normal patterns of inter-regional communication may contribute to cognitive problems in this group. The present magnetoencephalography study measured inter-regional phase synchronization while children with ASD and typically developing matched controls (6–14 years of age) performed a set-shifting task. Reduced theta-band phase synchronization was observed in children with ASD during extradimensional set-shifting. This reduction in task-dependent inter-regional connectivity encompassed numerous areas including multiple frontal lobe regions, and indicates that problems with communication among brain areas may contribute to difficulties with executive function in ASD.

## Introduction

Autism spectrum disorder (ASD) is associated with difficulties in cognitive development, particularly in domains such as social cognition and executive functions (Hill and Bird, 2006; O’Hearn et al., 2008). These abilities depend heavily on frontal lobe functions, and atypical frontal lobe development has been linked with cognitive difficulties in ASD (Courchesne and Pierce, 2005; Gilbert et al., 2008; Just et al., 2012). ASD is particularly associated with difficulties in cognitive flexibility, which includes the ability to switch between attentional or response sets (Yerys et al., 2009; Maes et al., 2011). This poor mental flexibility, the tendency to get “stuck” in a set is the underlying cognitive basis of the behavioral rigidity, perseveration, and repetitive behavior symptoms that are one of the definitive hallmarks of ASD (Hill, 2004). Several tasks have been used to assess cognitive flexibility behaviorally in ASD, most commonly being the Wisconsin Card Sorting Task (WCST), and have shown impaired performance for individuals with ASD on the WCST (e.g., Lopez et al., 2005). Evidence for cognitive flexibility impairment in ASD also comes from performance on the Cambridge Neuropsychological Test Automated Battery (CANTAB) Intradimensional-Extradimensional (ID-ED) Shift Task [developed by Dias et al. (1996)]. ED attentional shifts are thought to demand greater cognitive flexibility to successfully make the switch than ID shifts. Some studies have shown that individuals with ASD were impaired on ED shifts, but performed similarly to controls on ID shifts (Hughes et al., 1994; Ozonoff et al., 2004); however, other studies have failed to replicate this finding (e.g., Corbett et al., 2009). The neural basis of executive set-shifting abilities in ASD, however, remains unclear.

In previous neuroimaging studies atypical development of connectivity among cortical regions in ASD has been reported. Diffusion tensor imaging (DTI) studies have found that structural connections among cortical regions are atypical in ASD (see Travers et al., 2012 for review; Mak-Fan et al., 2013). Hemodynamic imaging has also demonstrated that functional connectivity among brain regions is abnormal in ASD, both in resting-state conditions and during the performance of cognitive tasks (see Müller et al., 2011 for review). Such results suggest that disordered development of brain network connectivity is common in autism and authors have suggested that this may lead to atypical interactions among brain regions resulting in social and cognitive impairments in this group (Just et al., 2007, 2012; Gilbert et al., 2008; Noonan et al., 2009).

The coordination of neural oscillations across brain areas is currently understood to underlie communication in distributed brain networks (Varela et al., 2001; Fries, 2005; Uhlhaas et al., 2009a). Neural coherence is related both to the organization of resting-state brain networks (Brookes et al., 2011; de Pasquale et al., 2012; Hipp et al., 2012) as well as to cortical network dynamics underling cognition and perception (see Palva and Palva, 2012 for review). Neural oscillations and their coherence develop throughout infancy, childhood and adolescence (Clarke et al., 2001; Uhlhaas et al., 2009b; Boersma et al., 2011, 2013a) and are relevant for the maturation of cognitive abilities (Benasich et al., 2008; Gou et al., 2011). Atypical oscillatory responses, including altered expression of local power and inter-regional coherence, have been reported in ASD using electroencephalography (EEG) and magnetoencephalography (MEG) (Sun et al., 2012; Wright et al., 2012; Khan et al., 2013). In resting-state networks atypical oscillatory synchrony has also been reported in ASD (Murias et al., 2007; Maxwell et al., 2013). Accumulating evidence that maturation of oscillatory activity in functional networks underlies cognitive development (see Uhlhaas et al., 2010 for review) highlights the importance of understanding the role of oscillatory neural synchronization during cognitive processing in neurodevelopmental disorders such as ASD.

Overall, the literature shows that frontal and parietal areas are implicated in tasks of cognitive flexibility, and there is some initial evidence for abnormalities in these areas in adults with ASD (Schmitz et al., 2006; Shafritz et al., 2008). However, to determine how these atypical patterns arise, investigation is needed in children with ASD. Previous findings have also suggested that abnormal coordination among brain regions may contribute to dysfunctional network interactions leading to cognitive difficulties in children with ASD (e.g., Just et al., 2007). In the present study, we recorded MEG while children with ASD and matched controls performed a set-shifting task requiring strong engagement of executive processes. In particular, this task places strong demands on cognitive flexibility. Atlas-based source reconstruction was performed and task-dependent changes in inter-regional oscillatory synchrony were assessed to test the hypothesis that children with ASD express atypical coordination of oscillatory activity in large-scale networks in tasks requiring cognitive flexibility and executive functions.

## Materials and Methods

### Subjects

A total of 20 children with ASD and 18 typically developing typically developing children were tested. However, due to too much movement during the MEG study, 4 in each group were excluded from analyses. Thus, 16 children with ASD (13 male; mean age 11.1 years; SD = 2.5) and 14 typically developing controls (12 male; mean age 11.5 years; SD = 2.4) were included in the present study. Intellectual ability was assessed using WASI 2-score IQ and did not differ between groups (*p* = 0.81); mean IQ was 107 (SD = 12.7) for the ASD group and 105.8 (SD = 15.6) for the controls. Subjects were excluded if they had a history of neurological disorders (other than ASD for those in the clinical group), brain injury, were using psychoactive medications, did not have normal or corrected-to-normal vision, an IQ below 75, language skill which made them unable to complete the task, or had other contraindications for MEG or MRI imaging. This research was approved by the Hospital for Sick Children Research Ethics Board. All children gave informed assent and their parents gave informed written consent.

### Task and data acquisition

**Figure 1:**
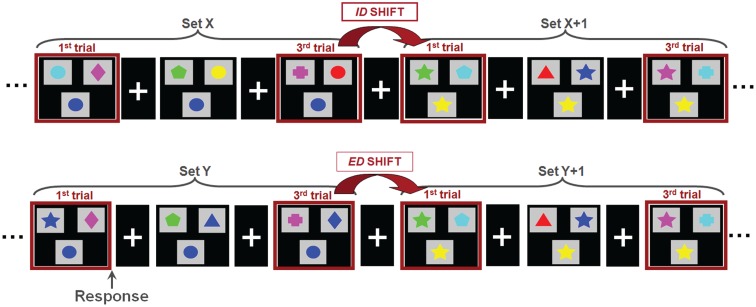
**The set-shifting paradigm**. Children were instructed to match the stimulus presented at the bottom of each image with one of two options presented at the top; the match was possible only by shape or color, always with only a single correct option. Stimuli were organized into sets, within which the rule for matching is held constant (i.e., green, star). Between sets, the rule for matching changed. In an intradimensional shifts (ID; top), the rule for matching switched within a dimension (i.e., within color; green to red) whereas in extradimensional shifts (ED; bottom) the rule for matching switched between dimensions (i.e., color to shape; green to star).

Magnetoencephalographic data were acquired on a 151 channel whole head CTF/MISL system (Coquitlam, Canada). Children were supine throughout data recording, and three coils placed at the nasion and left and right preauricular points were used to monitor head location during recording. Data were collected continuously at 625 Hz, during which subjects performed a set-shifting task (Figure 1). In this task, the children were presented with three stimuli on each trial. Subjects were instructed to match the bottom stimulus to one of the two upper stimuli. There were two dimensions on which the stimuli could vary: shape (triangle, circle, diamond, cross, star, pentagon) and color (cyan, blue, pink, green, yellow, red). The rule for correctly matching remained constant throughout each “set” and there was always only one correct response. Following three or four consecutive correct trials a “set-shift” occurred, in which the rule for matching would change. In ID shifts the rule would change within a given dimension (e.g., red to green, or star to cross); in ED shifts the rule for matching would change across dimensions, e.g., green to pentagon or triangle to yellow). The order of ID and ED shifts was randomized. Stimulus duration depended on subject response time, with a maximum duration of 4000 ms. The inter-trial interval varied between 1000 and 1500 ms. Two 6 min runs were collected for each subject. To facilitate co-registration of MEG activity to brain anatomy a structural volumetric MR image was collected following MEG recording for each subject using a 1.5-T Signa Advantage system (GE Medical Systems). MEG fiducial coils were replaced by radio-opaque markers for the T1 weighted 3-D SPGR scan to maintain accurate MEG-MRI co-registration.

### MEG preprocessing and source reconstruction

**Figure 2:**
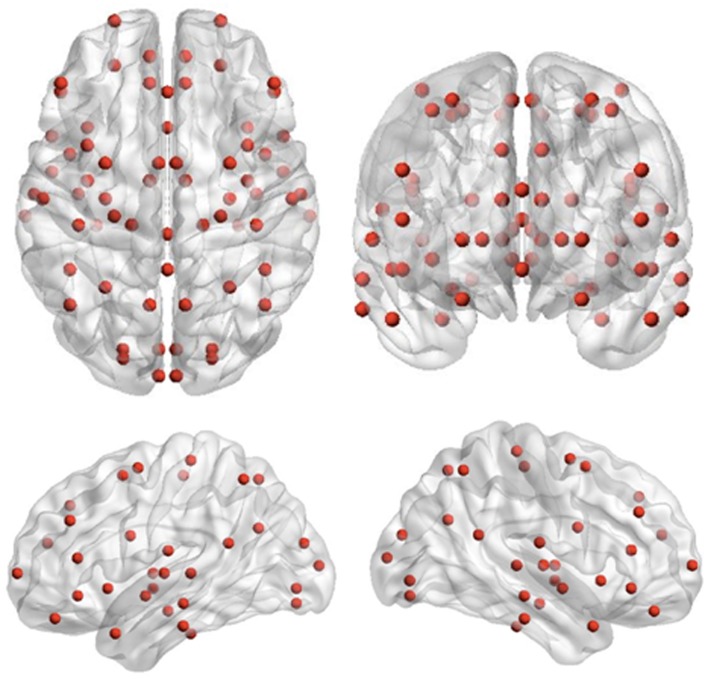
**Seed locations for the inter-regional phase-locking analysis**. Seed locations are the 72 cortical and sub-cortical regions originally adapted for source-space MEG analysis by Diaconescu et al. (2011).

Recording runs were excluded from analysis if more than 1 cm of movement occurred between head localization at the beginning and end of the run, consistent with movement thresholds for MEG studies in child populations (e.g., Pang, 2011; Taylor et al., 2011; Hung et al., 2012). Data epochs were extracted from −1500 to 2500 ms, relative to stimulus onset for trials immediately following extradimensional (ED1) and intradimensional (ID1) shift trials, as well as on the third trials of extradimensional (ED3) and intradimensional (ID3) sets, where task performance but no set-shifting was required. MEG data were co-registered with MRI data by aligning the fiducial markers, and a multisphere head model was constructed for each subject based on their individual MRIs. MRIs were normalized into standard MRI space using SPM2. Seed locations, which were 72 cortical and sub-cortical locations for source-space MEG analysis previously used by Diaconescu et al. (2011) (see Figure 2), were then unwarped from standard MRI space into the corresponding location in head space for each individual. The complete list of 72 locations, each Talairach coordinate and anatomical region can be found in Diaconescu et al. (2011) as well as Doesburg et al. (2013). Broadband time-series representing the activity of each of the 72 sources were then reconstructed for each trial using beamformer analysis. Beamformer analysis implements a spatial filter, estimating the activity at each location in the brain while maximally attenuating contributions from other sources, including ocular and muscle artifacts (Robinson and Vrba, 1999; Sekihara et al., 2001; Cheyne et al., 2006).

### Inter-regional phase-locking analysis

Data were filtered into theta (4–7 Hz), alpha (8–14 Hz), beta (15–30 Hz), low gamma (30–80 Hz), and high gamma (80–150 Hz) frequency ranges. Time-series of instantaneous phase values were then obtained for each source and frequency using the Hilbert transform. Inter-regional phase locking was calculated for each source pair and frequency using the phase lag index (PLI) which quantifies the stability of phase relations between source pairs across trials, while removing/attenuating phase locking occurring at zero/near-zero phase lag, thereby providing a measure of inter-regional phase synchronization that is robust against spurious synchronization originating from the activity of common sources (Stam et al., 2007). This strategy of performing phase-locking analysis on time-series of MEG activity reconstructed using beamformer analysis builds on established techniques for investigating oscillatory connectivity dynamics during cognitive processing (Doesburg and Ward, 2009; Doesburg et al., 2012b). This produced a 72-by-72 connectivity matrix for each time point and frequency, for each subject. Time-series of connectivity strengths for each region and frequency band were then calculated using the Brain Connectivity Toolbox (Rubinov and Sporns, 2010). This approach produces a graph metric for each region, for each analyzed time point. Connectivity strength accordingly indexes how functionally connected (phase-locked) a given region is to all other regions in the analyzed network. Time series of average connectivity strength were produced for each frequency by averaging across all 72 analyzed regions, and standardized relative to a −250 to 0 ms baseline interval to reveal the time courses of task-dependent changes in inter-regional phase locking for ED1, ID1, ED3, and ID3 conditions. This approach allowed the determination of dynamics in large-scale connectivity dynamics and the identification of time intervals for further statistical analysis. Time courses of mean connectivity strength averaged across 72 regions distributed across widely separated cortical and sub-cortical regions reflect fairly global coherence dynamics, whereas connectivity differences in children with ASD during the set-shifting task likely involve more specific subsets of connections, which we evaluated using the Network Based Statistic (NBS; see Zalesky et al., 2010). To this end, time windows exhibiting peaks in networks connectivity were selected, and connectivity matrices representing this task-dependent increase in network connectivity were obtained by averaging the connectivity matrices across time points in the peak window. A baseline connectivity matrix was constructed by averaging across an equivalent number of time points in the pre-stimulus interval. Task-dependent increases in connectivity were indexed by subtracting the baseline connectivity matrix from the active window connectivity matrix for each subject. Group differences in task-dependent phase locking were assessed using the NBS toolbox (Zalesky et al., 2010, 2012). This approach evaluates network differences between groups by first applying a univariate statistical threshold to each element in the connectivity matrix. If a connectivity component (a contiguous set of node pairs which are differentially connected between the compared groups) is discovered, its statistical significance is evaluated by shuffling the group membership, and observing the largest connectivity component observed in the shuffled data. Data surrogation was thus employed to create a null distribution, and the size of observed real connectivity components was considered relative to the surrogate data distribution to evaluate statistical confidence. As such, statistical significance is assigned at the level of the network connectivity component, rather than at the level of individual pair-wise comparisons (see Zalesky et al., 2010). As the largest components in the surrogate data are obtained considering all pair-wise comparisons, NBS controls for false positives due to multiple comparisons (Zalesky et al., 2010, 2012). NBS is a non-parametric technique that is appropriate for analysis of raw connectivity measures (see Zalesky et al., 2010), such as PLI. Seed regions and altered connectivity in children with ASD were plotted using the BrainNet Viewer toolbox (Xia et al., 2013).

## Results

### Behavioral performance on the set-shifting task

Behavioral data for children with ASD and typically developing controls are presented in Table 1. Reaction times for the typically developing control children were significantly longer (*p* < 0.02) on ED1 trials than for ED3 trials, but did not differ significantly between the ID1 and ID3 trials (*p* < 0.54). The children with ASD also exhibited significantly longer reaction times (*p* < 0.05) on ED1 trials than on ED3 trials, but did not express significantly longer RTs (*p* = 0.17) for ID1 relative to ID3 trials. Interestingly, children with ASD and controls did not differ in their reaction times, for either ED (ED1; *p* = 0.5) or ID set-shifting (ID1; *p* = 0.49). This indicates that the ED set-shifting task required additional cognitive resources for both the ASD and control groups, and overall performance did not differ between the groups.

**Table 1:** **Means and standard deviations of reaction times (ms) for children with ASD and typically developing controls for each trial type**.

	ASD	Controls
ED1	711 (77)	744 (165)
ED3	697 (68)	699 (141)
ID1	692 (82)	714 (88)
ID3	674 (72)	696 (130)

### Theta-band connectivity dynamics during set-shifting

**Figure 3:**
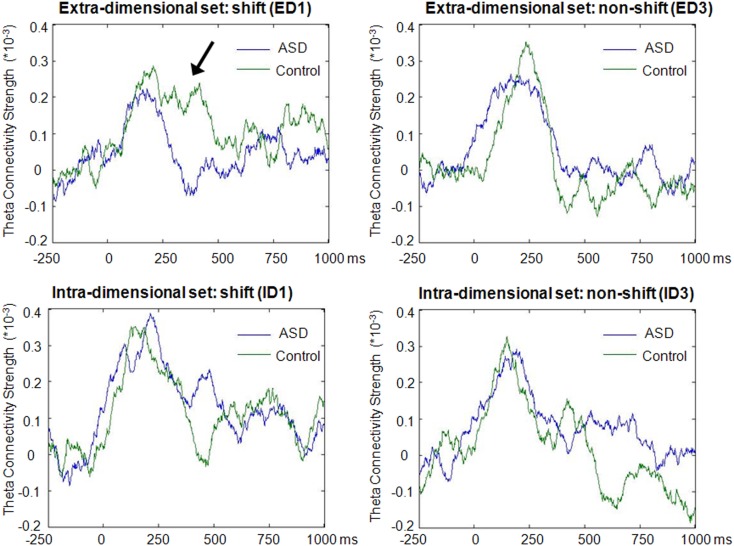
**Time-course of theta-band connectivity strength during task performance**. Time-courses of connectivity were derived by averaging theta-band (4–7 Hz) connectivity strengths across all 72 analyzed regions for each time point, and subtracting mean baseline (−250 to 0 ms) network connectivity from connectivity strength at each time point. Note the more extensive task-dependent increases in connectivity strengths in both groups during the first block of extradimensional (ED1) and intradimensional (ID1) sets, when increased engagement of executive functions was required, compared to the third trial of each set (ED3 and ID3) when set-shifting did not occur. These task-dependent modulations of network connectivity were observed for both groups, but reduced inter-regional theta connectivity was evident from 150 to 400 ms during extradimensional set-shifting (ED1) in children with ASD (arrow).

In the theta band, clear increases above baseline values of average network connectivity were seen for both children with ASD and typically developing controls in both the ID1 and ED1 shift conditions, as well as during ED3 and ID3 conditions, in which no set-shifting occurred. Figure 3 displays the time course of theta-band network connectivity strength, averaged across all 72 analyzed regions, for ASD and controls for each trial condition, standardized relative to a 250 ms pre-stimulus baseline. Time courses of task-dependent theta-band connectivity appeared roughly similar between ASD children and controls, except on ED1 shift trials, where demands on executive function are expected to be greatest. This reduced engagement of network connectivity in children with ASD, relative to typically developing controls, peaked 150–400 ms following stimulus onset. No clear modulations of inter-regional network synchronization were observed outside the theta frequency range.

### Reduced task-dependent inter-regional phase-locking in children with ASD

**Figure 4:**
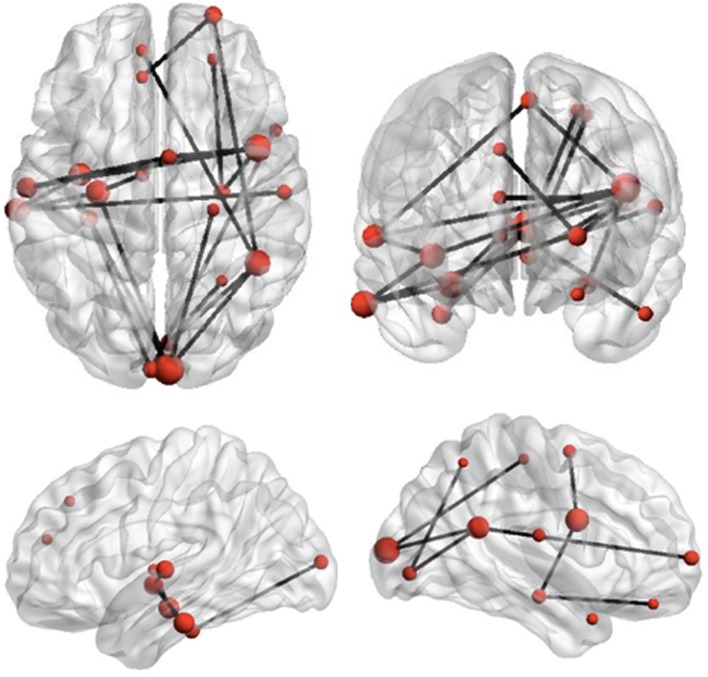
**Reduced network synchronization in ASD during set-shifting**. Black lines represent connections among regions which expressed reduced task-dependent theta-synchronization during extradimensional set-shifting (ED1) in children with ASD (*p* = 0.01). The size of the regions in this network, indicated by the size of the red dots, represents increasing magnitude of group differences in task-dependent connectivity strength for these regions.

The Network Based Statistic was employed to investigate group differences in theta connectivity during the ED1 set-shifting condition. Connectivity matrices reflecting network connectivity 150–400 ms post-stimulus, averaged across time points and bracketing the interval where group differences in connectivity were observed, were compared with connectivity matrices averaged across time points in a −250 to 0 ms pre-stimulus baseline, using a threshold of 2.5. This threshold is a *t*-statistic and is adapted for the data distribution being analyzed (see Zalesky et al., 2010, 2012). This revealed reduced task-dependent connectivity in children with ASD in a distributed network of brain regions which included strong involvement of frontal, temporal, and occipital brain regions, but also encompassed some sub-cortical and parietal brain areas (*p* = 0.01). Figure 4 displays the network expressing reduced theta-band connectivity in children with ASD, as well as the magnitude of group differences in regional connectivity strength for each region in this network. A complete list of each region in this network, together with its affiliated anatomical location and Talairach coordinates, is presented in Table 2. Comparison of connectivity between baseline and active windows within the ASD and typically developing groups, using NBS, did not reveal any statistically significant differences.

**Table 2:** **Each region in the network showing reduced connectivity in children with ASD, together with associated Brodmann areas (BA) and Talairach coordinates**.

Region	BA	*X*	*Y*	*Z*
Left medial prefrontal cortex	10	−8	48	20
Left dorsomedial prefrontal cortex	8	−8	36	40
Right orbitofrontal cortex	11	24	44	−20
Right frontal polar	10	24	64	4
Right medial premotor cortex	6	4	0	60
Right ventrolateral premotor cortex	9	44	4	24
Right primary motor cortex	4	24	−24	56
Left inferior temporal cortex	20	−64	−24	−24
Left ventral temporal cortex		−32	−28	−28
Left secondary auditory cortex	22	60	−14	4
Left parahippocampal cortex		−28	−16	−16
Right parahippocampal cortex		28	−16	−16
Right temporal pole	38	52	12	−28
Right inferior parietal cortex	40	44	−48	20
Right superior parietal cortex	7	28	−56	54
Right secondary somatosensory cortex	43	56	−16	16
Left secondary visual cortex		−4	−96	8
Right secondary visual cortex		4	−96	8
Right primary visual cortex		4	−84	−4
Left claustrum		−36	−8	−4
Left thalamus		−8	−8	4

## Discussion

Children with ASD expressed reduced theta-band network synchronization during ED set-shifting relative to typically developing controls. The RTs did not differ between the groups, indicating that they were matched on behavioral performance. Thus, differences seen in brain connectivity were not due to performance differences. In studies with adults with ASD, this was also found: despite no differences in behavior, fMRI results showed either increased (Schmitz et al., 2006) or decreased (Shafritz et al., 2008) activation patterns in widespread brain regions, linked to task performance. Our observation that performance differences were seen in the ED but not ID shifts is consistent with the ED task being more difficult and with the prior reports of greater difficulty in ASD with extra rather than ID shifts (e.g., Hughes et al., 1994; Ozonoff et al., 2004). Results from the present study also indicated that ED shifting (ED1 compared with ED3) resulted in significantly longer reaction times for both children with ASD and controls, whereas ID shifting did not. These findings are consistent with evidence indicating that clinical child populations can achieve comparable behavioral performance by recruiting different patterns of connectivity among brain areas, suggesting that brain systems associated with these functions in controls are impacted in ASD, leading to compensatory reorganization of function (e.g., Schafer et al., 2009).

Alterations in connectivity were widespread and encompassed frontal, parietal, occipital, temporal, and sub-cortical regions. These results add to a growing literature indicating that functional interactions among brain regions are atypical in ASD (Just et al., 2007; Murias et al., 2007; Müller et al., 2011; Sun et al., 2012; Boersma et al., 2013b). These alterations in functional connectivity may be related to abnormal development of underlying structural connections (see Travers et al., 2012 for review), including atypical inter-regional relations in cortical thickness (Shi et al., 2013). Altered structural brain connectivity has been associated with individual differences in cognitive ability in this group (Li et al., 2012). Recent studies, however, have shown that functional connectivity cannot be entirely predicted from brain structure (see Deco et al., 2011). This underscores the importance of understanding how functional interactions are expressed in ASD in the context of specific cognitive tasks. Several frontal regions were implicated in abnormal connectivity during set-shifting including prefrontal cortical areas. This is significant as ASD has been associated with atypical frontal lobe development (i.e., Ecker et al., 2012) as well as atypical connectivity between the frontal lobes and other regions of the brain (see Courchesne and Pierce, 2005). Viewed through this lens, our results suggest that disordered frontal lobe connectivity may impact the ability to marshal task-dependent functional interactions among frontal regions and other areas typically used to perform executive and cognitive tasks. Accordingly, disruptions of the frontal lobes’ capacity to orchestrate communication within distributed networks may contribute to selective difficulties in executive function in ASD.

There is a large body of work showing the importance of the prefrontal cortex in executive function in general and cognitive flexibility in particular. This evidence comes from lesion and neuropsychological studies, primate research, and neuroimaging studies. For example, patients with prefrontal lesions have difficulty on neuropsychological measures of flexibility such as the WCST (Stuss and Alexander, 2007). Seminal work by Dias et al. (1996) showed that damage to the dorsolateral prefrontal cortex of monkeys caused specific impairments in set-shifting. A host of neuroimaging studies have confirmed the importance of prefrontal cortex in cognitive flexibility, although specific regions have differed and also include parietal regions (e.g., Konishi et al., 1999; Dove et al., 2000; Sohn et al., 2000; Monchi et al., 2001; Zanolie et al., 2008). In light of this literature, the results of the present study may reflect reduced ability of prefrontal regions to coordinate activity among cortical regions to support set-shifting performance in children with ASD. Right ventrolateral premotor cortex also emerged as an important region in the pattern of reduced inter-regional connectivity during set-shifting in children with ASD, which is intriguing due to the proximity of this seed location to the right insula. Given results implicating right insula in executive abilities and the spatial resolution of MEG, it is possible this is due to reduced network engagement of right insula during ED set-shifting in children with ASD. Although the insula has been less frequently identified in reviews or meta-analyses of executive function, some studies have shown that the insula (specifically the right anterior insula) is functionally connected to other regions important for executive function, and may also play a role in monitoring of task performance. The right insula is part of a network hub responsible for switching between other brain systems during task performance (e.g., Sridharan et al., 2008; Eckert et al., 2009). In addition, a study of cognitive flexibility showed insula activation in a task switch condition (Dove et al., 2000), as well as a region in cuneus/precuneus that was more active with task switching. Accordingly, our findings suggest that reduced incorporation of right insula into distributed task-dependent networks may contribute to difficulties with cognitive flexibility and executive control in children with ASD.

Previous studies have also identified altered expressions of neural oscillations in ASD. This has included atypical local as well as long-range synchrony during cognitive and perceptual processing (i.e., Sun et al., 2012; Khan et al., 2013). Altered resting-state oscillatory coherence has been reported in ASD as well (Murias et al., 2007; Boersma et al., 2013b). Pertinent to the present study, altered long-range theta coherence has been described in ASD using EEG (Murias et al., 2007), and reduced task-dependent inter-regional phase-amplitude coupling has been reported in this population using MEG (Sun et al., 2012). Reduced ability to recruit long-range theta synchronization to support cognitive processing may be particularly pertinent for the ability of frontal regions to coordinate task-dependent activity in large-scale neuronal ensembles, as theta coherence has been proposed to play a primary role in long-distance communication among brain regions (von Stein and Sarnthein, 2000). This view is supported by findings relating long-range theta synchronization to the formation of task-dependent networks (Sarnthein et al., 1998; von Stein et al., 2000), as well as results indicating that theta rhythms are critical for the regulation of oscillations in other frequency ranges relevant for cognition, both within and across cortical regions (Schack et al., 2002; Canolty et al., 2006; Doesburg et al., 2012a,b). In view of this literature, our findings of reduced inter-regional theta-band phase synchronization in children with ASD suggest that inability to express task-dependent coordination of oscillatory coherence in large-scale functional brain networks may contribute to cognitive difficulties in this group. This model is consistent with the notion that inter-regional coherence of neuronal oscillations is a fundamental process which organizes information flow in the brain and supports cognition (Varela et al., 2001; Fries, 2005). Theta oscillations have been suggested to constitute a fundamental element of a “neural code” responsible for the organization of task-relevant information and its communication among brain areas (Lisman and Jensen, 2013). Our results add to growing evidence indicating that disruption of normal patterns of neural synchronization is related to functional impairments in numerous neurological and neuropsychiatric conditions, including neurodevelopmental disorders (Schnitzler and Gross, 2005; Uhlhaas et al., 2009a; Buzsáki and Watson, 2012).

Neural oscillations and their synchronization across brain regions have been proposed to play a key role in neurocognitive development (Uhlhaas et al., 2010). This is particularly pertinent for the present study, which demonstrates reduced inter-regional synchronization during an executive function task in children with ASD. Development throughout childhood and adolescence is associated with progressive shifts in the task-dependent expression of local oscillations (Clarke et al., 2001) and their long-range synchronization (Uhlhaas et al., 2009b). Individual differences in neuronal oscillations have also been related to the emergence of perceptual abilities (Csisbra et al., 2000) and the development of cognitive abilities (Benasich et al., 2008; Gou et al., 2011). Atypical oscillatory synchronization in brain networks has been related also to functional impairments and cognitive difficulties in other pediatric populations (Doesburg et al., 2011; Ibrahim et al., 2012). These age-dependent changes are understood to reflect changes in the architecture of functional brain networks, reflecting maturation in pathways of information flow in the brain (Boersma et al., 2011, 2013a).

## Conclusion

This study provides the first evidence for reduced theta-band inter-regional synchronization during a set-shifting task in children with ASD. This adds to the growing body of literature demonstrating disrupted oscillatory coherence in brain networks in neurodevelopmental disorders. The inability to recruit theta-band synchronization in large-scale networks may contribute to deficits in executive abilities associated with ASD. This altered connectivity included several frontal regions, and including the right insula, suggesting that frontal lobe functions are less effective in coordinating among task-relevant brain regions in ASD, leading to reduced performance on tasks requiring executive abilities.

## Conflict of Interest Statement

The authors declare that the research was conducted in the absence of any commercial or financial relationships that could be construed as a potential conflict of interest.
